# Serum Concentrations and Gonadal Expression of INSL3 in Eighteen Males With 45,X/46,XY Mosaicism

**DOI:** 10.3389/fendo.2021.709954

**Published:** 2021-08-10

**Authors:** Marie Lindhardt Ljubicic, Anne Jørgensen, Lise Aksglaede, John Erik Nielsen, Jakob Albrethsen, Anders Juul, Trine Holm Johannsen

**Affiliations:** ^1^Dept. of Growth and Reproduction, Rigshospitalet, University of Copenhagen, Copenhagen, Denmark; ^2^International Center for Research and Research Training in Endocrine Disruption of Male Reproduction and Child Health (EDMaRC), Rigshospitalet, University of Copenhagen, Copenhagen, Denmark

**Keywords:** INSL3, 45,X/46,XY, LC-MS/MS, gonadal histology, immunohistochemistry

## Abstract

**Objective:**

Insulin-like factor 3 (INSL3) is produced in the testes and has been proposed as a circulating biomarker of Leydig cell capacity, but remains undescribed in 45,X/46,XY mosaicism. The aim was to examine serum concentrations and gonadal expression of INSL3 in 45,X/46,XY mosaicism.

**Methods:**

Retrospectively collected data from medical records, gonadal tissue samples, and prospectively analyzed serum samples from eighteen male patients with 45,X/46,XY mosaicism (one prepubertal, four testosterone-treated, 13 untreated) were included. Biochemical, clinical, and histological outcomes were evaluated according to serum INSL3 concentrations, quantified by LC-MS/MS methodology, and gonadal INSL3 immunohistochemical expression.

**Results:**

Serum INSL3 concentrations spanned from below to above the reference range. In untreated patients, the median serum INSL3 SD score was -0.80 (IQR: -1.65 to 0.55) and no significant difference was observed between INSL3 and testosterone. There was no clear association between serum INSL3 and External Genitalia Score at diagnosis, spontaneous puberty, or sperm concentration. INSL3 and CYP11A1 expression overlapped, except for less pronounced INSL3 expression in areas with severe Leydig cell hyperplasia. No other apparent links between INSL3 expression and histological outcomes were observed.

**Conclusions:**

In this pilot study, serum INSL3 concentrations ranged and seemed independent of other reproductive hormones and clinical features in males with 45,X/46,XY mosaicism. Discordant expression of INSL3 and CYP11A1 may explain low INSL3 and normal testosterone concentrations in some patients. Further studies are needed to elucidate the divergence between serum INSL3 and testosterone and the potential clinical use of INSL3.

## Introduction

Insulin-like factor 3 (INSL3) is a testicular peptide hormone produced by Leydig cells, which has been proposed as a new marker of testicular function. Studies have suggested that testosterone and INSL3 provide different information about Leydig cell function in hypogonadal patients (1–3) and that INSL3 may play a role in ensuring germ cell survival *via* paracrine pathways in the seminiferous tubules in male rats (4, 5). In general, INSL3 is considered a circulating marker that reflects Leydig cell number and differentiation status (6–8), but the exact clinical use of INSL3 quantification remains elusive. Testicular expression of INSL3 has been described in several studies, including as an indicator of mature Leydig cells (9), declining with increasing age (10), and in dysgenetic testes from patients with Klinefelter syndrome (11).

Patients with a 45,X/46,XY mosaic karyotype and male sex of rearing present with varying phenotypes both in terms of clinical findings and gonadal histology. The clinical presentation ranges from phenotypic males to phenotypic females. Regardless of phenotype at birth, most male patients with 45,X/46,XY enter puberty spontaneously (12–16), but may subsequently need testosterone replacement therapy (12, 17, 18) due to varying degrees of primary testicular dysgenesis (12, 16). Thus, serum concentrations of testosterone vary from low to normal, while concentrations of inhibin B often are low (13–15). Gonadal histology similarly spans from streak gonads to near-normal testicular tissue with complete spermatogenesis in some tubules (12, 19–22). Leydig cell hyperplasia and Sertoli-cell-only (SCO) pattern are frequent findings in testicular samples although SCO is often seen focally (12, 16, 20). Additionally, males with 45,X/46,XY mosaicism are often infertile, but reports of patients with spermatozoa in the ejaculate or complete spermatogenesis in histological samples exist (12, 17, 23–25). Lastly, patients with 45,X/46,XY mosaicism are characterized by an increased risk of developing gonadal neoplasia with the malignant precursors of germ cell neoplasia *in situ* or gonadoblastoma (12, 16). Thus, the clinical management of this heterogeneous group of patients can be a challenge in terms of gonadal function, possible need of testosterone treatment, surveillance due to the risk of germ cell malignancy, and impaired fertility potential. A new biomarker of clinical and/or histological outcomes could therefore potentially guide clinical management in these patients.

Hitherto, serum concentrations and expression in gonadal tissue of INSL3 from patients with 45,X/46,XY mosaicism remain undescribed. Therefore, in this pilot study of male patients with 45,X/46,XY mosaicism, we aimed to evaluate serum INSL3 concentrations and immunohistochemical expression of INSL3 in gonadal tissue from biopsies and gonadectomies along with clinical and histological outcomes, respectively.

## Material and Methods

### Study Population

In total, 18 male patients with 45,X/46,XY mosaicism followed at the Department of Growth and Reproduction, Copenhagen, were included. The inclusion criteria were: 1) a 45,X/46,XY karyotype including variants; 2) male sex of rearing; and 3) a planned visit at the outpatient clinic during one of two time periods (February to November 2018 or February to November 2020) at which a blood sample would be drawn. Patients with variants of the cell lines other than 45,X/46,XY (such as derivative Y chromosomes) and a single patient with a karyotype of 45,X/46,XX-*SRY* positive were also included. Two patients with expected visits were included and the visits were subsequently cancelled. For these patients, no blood samples were available for analysis in the present study, but their clinical and histological data were included. In all patients, the karyotype was verified through medical records. Clinical data extracted from medical records included information on; 1) external genital phenotype at time of diagnosis, scored according to the External Genitalia Score (EGS) ranging from 0-12 points, with increasing scores indicating increasing degrees of androgenization (26); 2) spontaneous pubertal onset (yes, no); 3) type of testosterone treatment (transdermal, intramuscular, none) if the patient was substituted with testosterone at the time of blood sampling; 4) gonadal surgery (biopsy, gonadectomy); and 5) semen analysis (azoospermia, concentration).

Clinical, biochemical, and/or histological features on nine of the 18 included patients have previously been published (12, 13), but serum INSL3 concentrations and immunohistochemical stainings are new and uncovered aspects.

### Gonadal Immunohistochemistry and Histological Descriptions

Data on possible gonadal biopsy and/or gonadectomy, including previous pathology reports, was retrieved for each patient when available. All gonadal samples derived from surgery solely due to clinical diagnostics or management. The histological material was independently described by two specialists in gonadal histopathology (AJø and LA). These descriptions of overall morphology and presence of different cell types were based on sample availability in the following, preferred order: 1) newly hematoxylin-eosin (HE)-stained tissue sections from stored tissue blocks; 2) older images saved electronically; or 3) previous pathology reports.

In the nine patients with stored tissue blocks, immunohistochemical staining for HE, INSL3,CYP11A1, and 3β-HSD was carried out as follows: gonadal tissue was fixed in Stieve or modified Stieve fixative followed by dehydration and paraffin embedding according to standard procedures. Serial sections (4 µm) were dewaxed and rehydrated prior to immunohistochemistry (IHC). Tissue sections were subjected to heat-induced antigen retrieval buffer by microwaving in citrate buffer (10 mM, pH 6.0). Blocking of endogen peroxidase activity and reduction of non-specific antibody binding was done by incubation in 0.5% (v/v) H_2_O_2_ in H_2_O for 30 minutes, followed by incubation in 0.5% skimmed milk in PBS for 30 minutes. Tissue sections were washed in Tris-buffered saline (TBS) between protocol steps except between horse serum PBS/BSA blockade and primary antibody. All incubations were carried out in a humidity box. Primary antibodies were diluted 1:1500 (INSL3, HPA028615) and 1:250 (CYP11A1, HPA016436) (Atlas AB, Sweden) in TBS and sections were incubated overnight at 4° C, followed by 1 hour at room temperature. Immunohistochemical staining for 3β-HSD was carried out by subjecting sections to heat-induced antigen retrieval in a pressure cooker in TEG buffer (10 mM Tris, 0.5 mM EGTA, pH 9.0). Blocking of endogen peroxidase activity peroxidase and reduction of non-specific antibody binding was done by incubation in 1% (v/v) H_2_O_2_ in MeOH for 30 minutes, followed by incubation in horse serum (20% v/v) with PBS/BSA (5% w/v) ImmPRESS (Vector Laboratories, Burlingame, California) for 30 minutes. Tissue sections were washed in Tris-buffered saline (TBS) between protocol steps except between horse serum PBS/PSA blockade and primary antibody, and all incubations were carried out in a humidity box. The primary antibody was diluted 1:100 (3β-HSD, HPA043261) in horse serum PBS/BSA, and sections were incubated overnight at 4°C, followed by 1 hour at room temperature. For both protocols, sections were subsequently incubated for 30 minutes with antirabbit-horse radish peroxidase secondary antibody (MP-7401, Vector Laboratories, Burlingame, California). Visualization was performed using ImmPACT AEC peroxidase substrate (Vector Laboratories, Burlingame, California). Counterstaining with Mayer’s hematoxylin was done before mounting with Aquatex (Merck, Darmstadt, Germany). Negative controls were included and processed with the primary antibody replaced by the dilution buffer alone, none of which showed staining. Sections were initially evaluated on a Nikon Microphot-FXA microscope and then by scanning slides on a NanoZoomer 2.0 HT (Hamamatsu Photonics, Herrsching am Ammersee, Germany).

Leydig cell hyperplasia was quantified by determining the “Total Leydig Cell Area (TLCA)/Total Selected Area (TSA)” as described by Tarsitano et al. with a few modifications (27). In brief, quantification was conducted on the sections stained for CYP11A1 expression and the entire area of the testicular biopsy was included, i.e. not a selected area. The area of every single Leydig cell group was manually delineated on the sections using the NDPview version 1.2.36 software (Hamamatsu Photonics) and the TLCA was calculated by adding up the areas of all the Leydig cell groups. Thus, TLCA/TSA represents the percentage of the entire biopsy that is made up by Leydig cells.

### Hormone Assays

Biochemical data was obtained from the blood samples drawn as part of routine follow-up. Samples were taken either in untreated patients (n=14) or after the initiation of testosterone treatment (n=4). Serum concentrations of luteinizing hormone (LH), follicle-stimulating hormone (FSH), testosterone, sex hormone-binding globulin (SHBG), anti-Müllerian hormone (AMH), and inhibin B were analyzed as per routine when requested by the treating physician immediately following sampling. Subsequently, INSL3 was analyzed in all samples after a maximum storage of three months at -20° C. Lastly, in case of missing analyses (due to lack of request by treating physician), these were performed and included FSH, AMH, and inhibin B.

Serum INSL3 was quantified by liquid chromatography-tandem mass spectrometry (LC-MS/MS) as described previously (28). The limit of detection (LOD) was 0.03 µg/L, and the inter-assay coefficient of variation (CV) was below 9%. LH and FSH concentrations were quantified by a time-resolved fluoro-immunometric assay (AutoDELFIA, Perkin Elmer, Turku, Finland) with LODs of 0.05 IU/L and interassay CVs below 5% for both. Serum testosterone concentrations were analyzed by either a chemiluminescence immunoassay (Access 2, Beckman Coulter, Brea, CA, USA) or by LC-MS/MS as previously described (29). LODs and CVs were 0.35 nmol/L and below 5% (Access 2), and 0.10 nmol/L and below 6% (LC-MS/MS). All Access-based testosterone measurements were factorized to LC-MS/MS levels based by an internal method comparison. Serum concentrations of SHBG and AMH were analyzed by Access 2 with LODs of 0.35 nmol/L and 0.14 pmol/L, respectively, and CVs below 6% and 5%, respectively. Serum concentrations of Inhibin B were measured by an enzyme-linked immunosorbent assay (Beckman Coulter Inhibin B Gen II ELISA, Beckman Coulter, Brea, CA, USA) with an LOD of 3 pg/mL and a CV below 11%. The Danish Accreditation Fund for medical examination accredited all analyses according to the standard DS/EN 15189.

Hormone concentrations were plotted against previously published reference ranges (3, 30, 31). All measurements below any given LOD were plotted as *LOD/2*.

### Statistical Methods

All data was reported as medians and interquartile ranges due to the small sample size. Correlations were evaluated using Spearman’s rho. P-values below 0.05 were considered statistically significant. All analyses were performed using IBM Statistics SPSS, version 25. Based on previously published normative ranges (3, 30, 31) established using the Generalized Additive Model for Location, Scale and Shape (GAMLSS), measurements for INSL3, testosterone, and LH were converted to standard deviation (SD) scores. SD scores were calculated as follows: SD score = ((X/M)^L^-1)/(L×S), where X is the measurement and L ≠ 0. SD scores allowed for comparison of hormone values across all ages.

## Results

### Serum INSL3 Concentrations and Clinical Outcomes

Information on the 18 male patients with 45,X/46,XY mosaicism aged 8.4 to 60.9 years is shown in **Table 1**. EGS at birth ranged from 2 to 12 with a median of 12. There was no correlation between INSL3 SD scores later in life and EGS at birth in the untreated, post-pubertal patients (r^2^ = 0.20, p=0.52, **Table 1**). Thirteen patients were biopsied or gonadectomized. All patients older than nine years of age experienced spontaneous pubertal onset, except for the patient who was bilaterally gonadectomized at the age of 14 years. There was no association between serum INSL3 SD scores later in life and previous spontaneous pubertal onset in the untreated, postpubertal patients given that all untreated patients older than 8 years of age entered puberty spontaneously despite varying INSL3 SD scores. Three out of 16 postpubertal patients with at least one intact testis (aged 40.7 to 60.9 years) received testosterone replacement therapy (two transdermal and one intramuscular).

**Table 1:** Serum concentrations of insulin-like factor 3 (INSL3) and clinical findings in 18 males with 45,X/46,XY mosaicism.

ID	Age^1^	INSL3 (ug/L)	INSL3 (SDS)	T Treatment^2^	Spont pub	External Genitalia Score^3^						Semen analysis (mio/mL)	Biopsy/ gonadectomy	
						*Labioscrotal fusion*	*Genital tub length (mm)*	*Urethral meatus*	*Right gonad*	*Left gonad*	*Total*		*L*	*R*
**1**	8.4	<LOD	0.7	none	NA	3	2.5	2	1.5	0	9	NA	B	B
**2**	12.7	0.39	0.1	none	yes	3	3	3	1.5	1.5	12	NA	–	–
**3**	13.1	0.28	-0.6	none	yes	3	3	3	1	1.5	11.5	NA	B	B
**4**	13.5	0.26	-1.0	none	yes	3	3	3	1.5	1.5	12	NA	–	–
**5**	14.8	0.83	-0.1	none	yes	3	2.5	3	1.5	1.5	11.5	NA	–	–
**6**	16.7	0.42	-1.8	none	yes	3	3	3	1.5	1.5	12	NA	–	–
**7**	20.1	3.33	2.7	none	yes	3	3	3	1.5	1.5	12	crypto	B	B
**8**	21.3	<LOD	-6.5	i.m.	no	1.5	0	0	0.5	0	2	–	G	G
**9**	29.1	0.68	-1.5	none	yes	3	2.5	1	0	1.5	8	11	B	G
**10**	31.0	–	–	none	yes	3	3	3	1.5	1.5	12	0.06	G	B
**11**	31.9	–	–	none	yes	3	1.5	2	0	1.5	8	–	B	G
**12**	38.1	0.75	-1.1	none	yes	3	3	2	1.5	1.5	11	azoo^a^	B	B
**13**	40.7	0.54	-1.7	none	yes	3	3	3	1.5	1.5	12	azoo^a^	B	B
**14**	40.8	0.26	-3.0	T.D.	yes	3	3	3	1.5	1.5	12	azoo^a^	B	B
**15**	46.2	<LOD	-5.8	i.m.	yes	3	3	3	0.5	1.5	11	azoo^b^	B	–
**16**	50.7	0.50	-1.8	none	yes	3	3	2.5	0.5	1.5	10.5	azoo^b^	B	B
**17**	55.4	2.63	2.0	none	yes	3	3	3	1.5	1.5	12	azoo^a^	–	–
**18**	60.9	0.04	-4.9	T.D.	yes	3	3	3	1.5	1.5	12	azoo^a^	B	B

T, testosterone; Spont. pub., spontaneous puberty; Genital tub. length, genital tubercle length; LOD, limit of detection; NA, not applicable due to age; i.m., intramuscular injection; T.D., transdermal; prepub, prepubertal; B, biopsy; G, gonadectomy; crypto, cryptozoospermia; azoo, azoospermia; ^1^Age at the time of blood sampling for INSL3 analysis; ^2^Testosterone-treatment at the time of blood sampling; ^3^External Genitalia Score at first presentation; a non-obstructive azoospermia; described in patient file, sample not analyzed at Dept. of Growth and Reproduction.

### Serum INSL3 Concentrations and Other Reproductive Hormones

**Figure 1:**
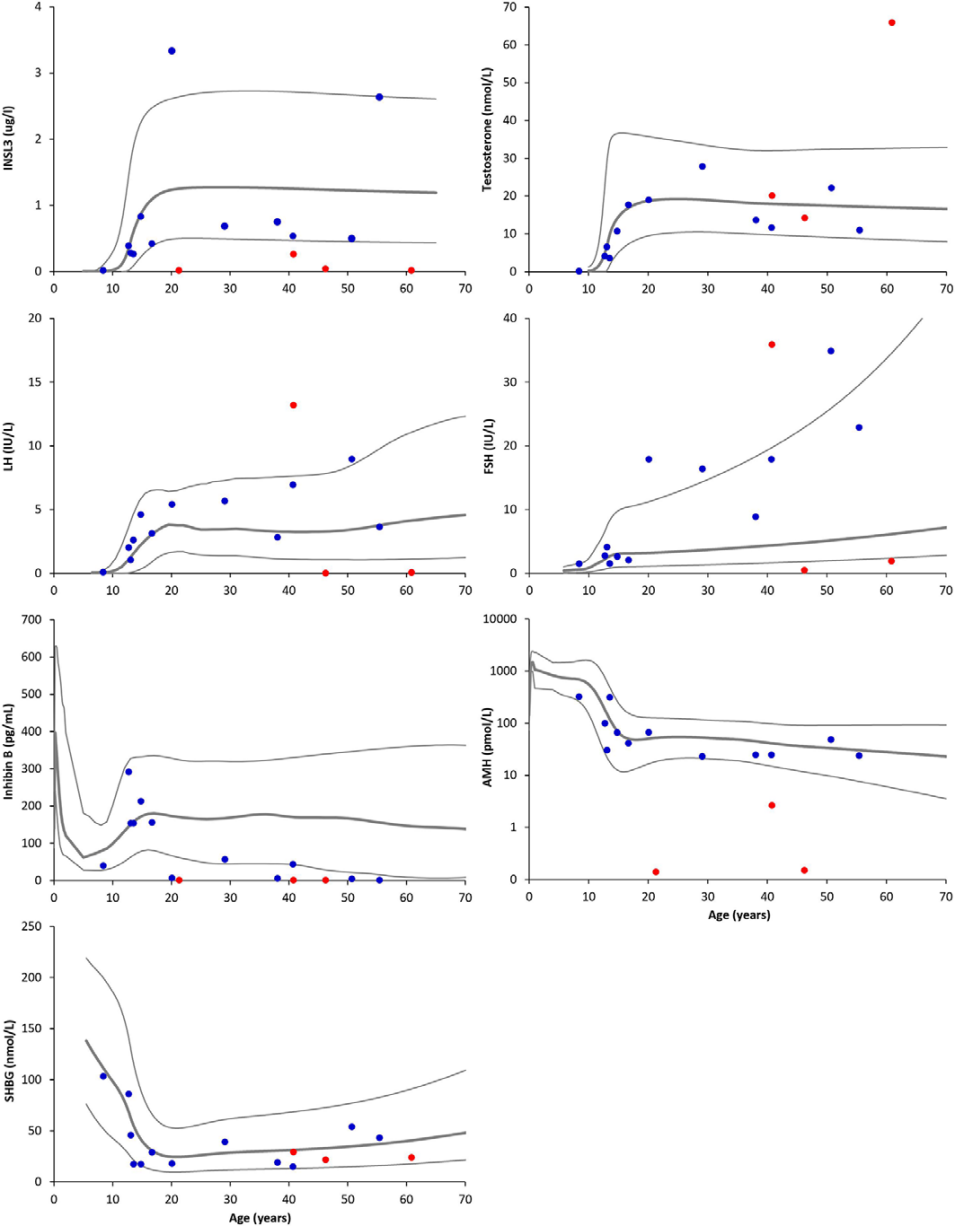
Serum concentrations of insulin-like factor 3 (INSL3), testosterone, luteinizing hormone (LH), follicle-stimulating hormone (FSH), inhibin B, anti-Müllerian hormone (AMH, note: logarithmic Y axis), and sex hormone-binding globulin (SHBG) according to age in male patients with 45,X/46,XY mosaicism. Red dots indicate testosterone-treated patients, while blue dots indicate untreated patients. Grey lines indicate +2 SD, mean, and -2 SD of the respective reference ranges.

For the total study population, serum INSL3 concentrations ranged from <LOD to 3.33 µg/L (**Table 1** and **Figure 1**). In the untreated patients, the median serum INSL3 SD score was -0.80 (IQR: -1.65 to 0.55, range: -1.8 to 2.7). The INSL3 concentrations were below LOD in a prepubertal patient, and in three patients receiving testosterone treatment. Notably, all patients with INSL3 SD scores below -2 (n=4) were undergoing testosterone treatment (**Table 1**).

**Figure 2:**
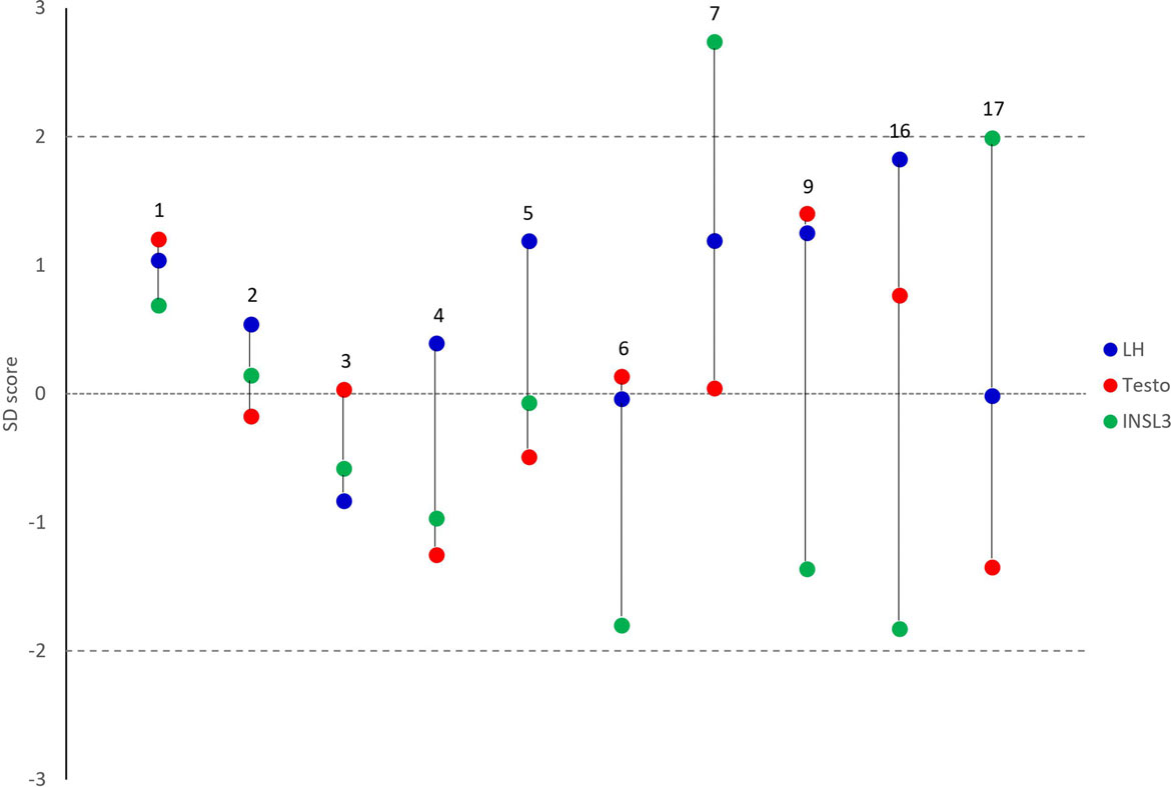
Measurements of insulin-like factor 3 (green), Luteinizing hormone (blue), and testosterone (red) expressed as standard deviation (SD) scores in untreated male patients with 45,X/46,XY mosaicism. Numbers indicate IDs corresponding to numbers in **Tables 1**, **2**. Dashed lines represent +/-2 SD scores, while the dotted line represents the mean.

While serum INSL3 concentrations spanned across the entire normative range, and above and below, serum testosterone concentrations were low-normal to normal (**Figure 1**), whereas LH and FSH mostly ranged from normal to high. Inhibin B and AMH concentrations were low to normal, while serum SHBG concentrations were within the normal range for almost all patients. When comparing SD scores for INSL3 and testosterone in the untreated patients, there was no significant difference with medians of -0.8 and 0.04, respectively (**Figure 2**). However, in nearly half of the untreated patients, LH was the highest of the three hormones (**Figure 2**).

### Serum INSL3 Concentrations and Semen Analyses

Seven of ten patients with available semen analyses were azoospermic (**Table 1**). In three patients, semen sampling revealed concentrations of 0.06, 11 mio/mL, and cryptozoospermia (i.e. few spermatozoa present in the centrifuged pellet), respectively. No significant difference was observed between INSL3 SD scores in untreated patients with azoospermia (INSL3 SD scores ranged from -1.1 to +2, n=4) and with spermatozoa (INSL3 SD scores of -1.5 and +2.7, n=2, p = 0.53, **Table 1**).

### Gonadal INSL3 Expression and Histological Outcomes

Gonadal histological descriptions from biopsy and/or gonadectomy were available in 13 of the 18 included patients (**Table 2**). None of the postpubertal samples revealed normal testicular morphology (n=21). In 68% of post-pubertal samples, Leydig cell hyperplasia was present (n=13/19), while Sertoli cell-only pattern was focally or universally present in 79% (n=15/19). Germ cells were present in 40% of samples (n=8/20) with spermatocytes as the most differentiated germ cell stage observed. Discrepancies in the total number of biopsies/gonadectomies analyzed were due to differences in available information. In 15% of patients with available histological material (n=2/13), germ cell neoplasia *in situ* was observed.

**Table 2:** Serum insulin-like factor 3 (INSL3) concentrations and histological findings in 13 males with 45,X/46,XY mosaicism.

ID	INSL3 (SDS)	EGS	B or G: age (yrs)	Overall morphology	*Histological characteristics*						Semen analysis	*IHC*			TLCA/TSA	
					Testicular tubules	Leydig cells	SCO	Female charact.	Germ cells	Germ cell types/spermatogenesis		CYP11A1	INSL3	3β-HSD		
**1**	0.7	9	G (l): 1.4	prepubertal testis	normal	NA due to age	n	n	present	spermatogonia	NA due to age					
			B (r): 0.5	prepubertal testis	normal	NA due to age	n	n	present	spermatogonia						
**3**	-0.6	11.5	B (l): 6.2	prepubertal testis	NA due to age	NA due to age	n	n	present	pre-spermatogonia	NA due to age					
**7**	2.7	12	B (l): 19.1	dysgenetic testis	normal	hyperplasia	y	n	absent	–	azoo	+++	+++	++		18%
			B (r): 19.1	dysgenetic testis	normal	hyperplasia	y	n	absent	–		+++	+++	++		19%
**8**	-6.5	2	G (l): 2.2	fibrotic ovarian-like stroma	NA	NA	NA	y	absent	–	NA due to age	÷	÷	÷		0%
			B (r): 2.2	prepubertal testis	normal	present	y/n	n	present	spermatogonia and gonocytes/GCNIS						
			G (r): 14.2	dysgenetic testis	normal	present	n	n	present	GCNIS, no non-malignant cells, no spermatogenesis		+	+	÷		<1%
**9**	-1.5	8	B (l): 2.8	prepubertal testis	normal	undiff.	n	n	present	spermatogonia	NA due to age	+	÷	÷		3%
			G (r): 2.8	no testicular tissue	NA	NA	NA	n	NA	–		÷	÷	÷		0%
			B (l): 24.8	dysgenetic testis	thick basal membrane	hyperplasia	y	n	present	spermatogonia and spermatocytes	11 mio/mL	+++*	+++*	+		27%
**10**	–	12	B (l): 28.7	dysgenetic testis	thick basal membrane, fibrosis	slight hyperplasia	n	n	present	GCNIS, no spermatogenesis	0.06 mio/mL					
			B (r): 28.7	slightly dysgenetic testis	slightly thick basal membrane	normal	n	n	present	spermatogonia and spermatocytes						
			G (l): 29.8	dysgenetic testis	thick basal membrane, some hyalinized	normal	n	n	present	GCNIS, no non-malignant cells, no spermatogenesis		+++	+++	++		12%
**11**	–	8	B (l): 14.0	no gonadal tissue	NA	NA	NA	NA	NA	–		÷	÷	÷		0%
			G (r): 14.0	ovotest. remnant	present	–	–	ovotest. remnant	absent	–						
			B (l): 21.3	dysgenetic testis	thick basal membrane, some hyalinized	hyperplasia	y/n	n	present	spermatogonia and spermatocytes		+++*	+++*	++		30%
**12**	-1.1	11	B (l): 30.1	dysgenetic testis	thick basal membrane, some hyalinized	normal	y	n	absent	–	azoo	+++	+++	+		15%
			B (r): 30.1	dysgenetic testis	thick basal membrane, some hyalinized	slight hyperplasia	y	n	absent	–		+++	+++	+		12%
**13**	-1.7	12	B (l): 28.2	dysgenetic testis	thick basal membrane	normal	y	n	absent	–	azoo	++	++	+		14%
			B (r): 28.2	dysgenetic testis	thick basal membrane	normal	y	n	absent	–		+++	+++	+		17%
**14**	-3.0	12	B (l): 39.5	dysgenetic testis	very thick basal membrane	hyperplasia, nodules	y	n	absent	–	azoo	+++*	+++*	++		43%
			B (r): 39.5	dysgenetic testis	very thick basal membrane	hyperplasia, nodules	y	n	absent	–		+++*	+++*	++		55%
**15**	-5.8	11	B (l): 28.2	dysgenetic testis/streak gonad	fibrotic basal membranes	severe hyperplasia, large nodules	y	y (streak)	absent	–	azoo					
**16**	-1.8	10.5	B (l): 47.5	dysgenetic testis	atrophic and hyalinized basal membranes	hyperplasia	y	n	absent	–	azoo					
			B (r): 47.5	dysgenetic testis	atrophic and hyalinized basal membranes	hyperplasia	y	n	absent	–						
**18**	-4.9	12	B (l): 36.3	dysgenetic testis	thick basal membrane	hyperplasia	y/n	n	present	spermatogonia	azoo	+++	+++	++		21%
			B (r): 36.3	dysgenetic testis	thick basal membrane	hyperplasia	y/n	n	present	spermatogonia		+++	+++	++		19%

EGS, external genitalia score; B, biopsy; G, gonadectomy; yrs, age at biopsy/gonadectomy in years; SCO, Sertoli cell-only pattern; charact., characteristics; l, left; r, right; LOD, limit of detection; y, yes; n, no; NA, not applicable; Undiff., undifferentiated; GCNIS, germ cell neoplasia in situ; ovotest. remnant, ovotesticular remnant; azoo, azoospermia; IHC, immunohistochemical staining; ÷, Leydig cell (LC) negative; +, few positive LCs; ++, many positive LCs; +++, all LCs positive, except in areas with severe LC hyperplasia where * indicates incomplete overlap in INSL3 and cytochrome P450 Family 11 Subfamily A Member 1 (CYP11A1) expression with a sub-population of INSL3-negative and CYP11A1-positive LCs; 3β-HSD, 3β-hydroxysteroid dehydrogenase; TLCA, total Leydig cell area; TSA, total selected area (entire biopsy area).

**Figure 3:**
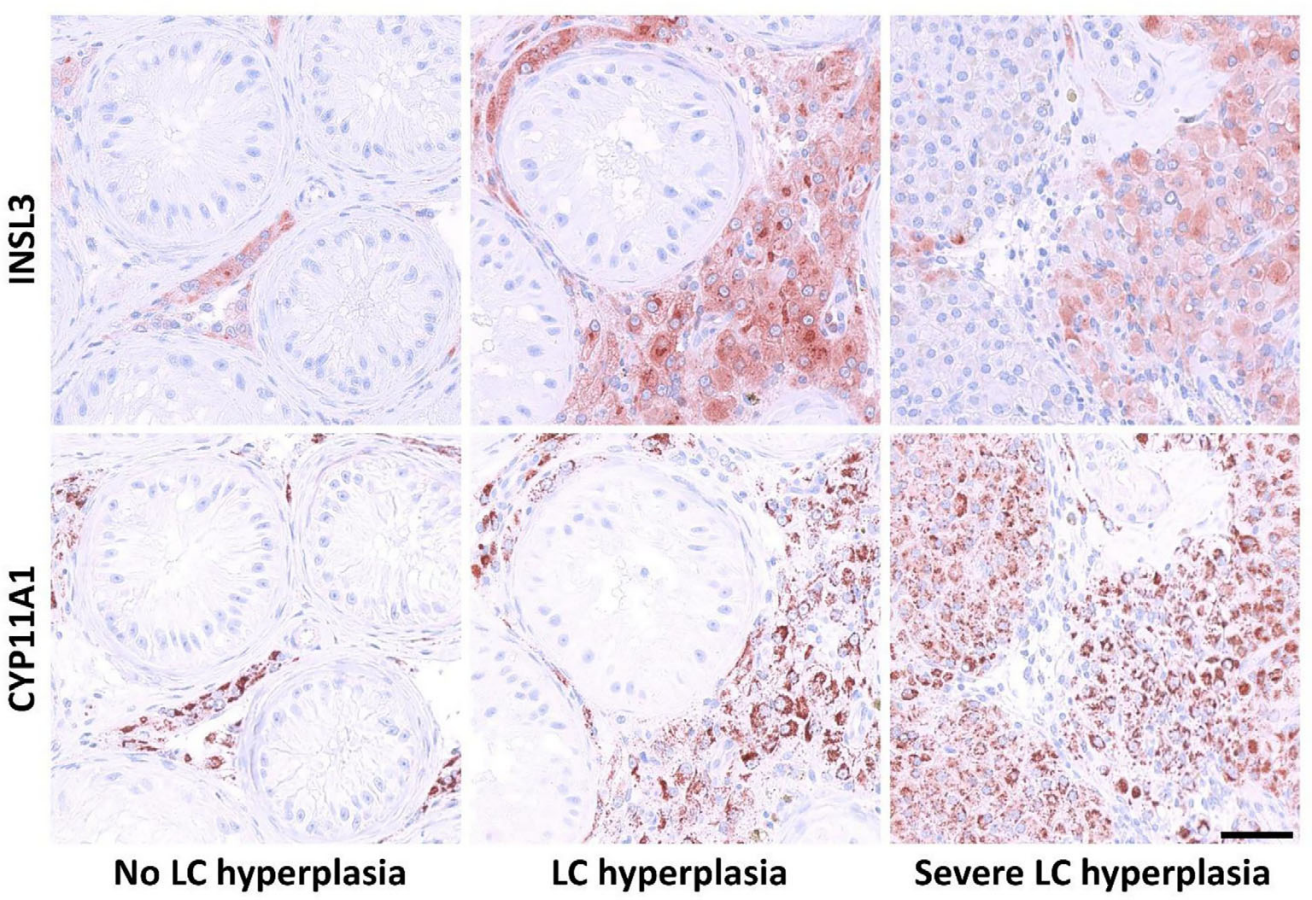
Expression pattern of Insulin-like factor 3 (INSL3) and cytochrome P450 Family 11 Subfamily A Member 1 (cholesterol side-chain cleavage enzyme, CYP11A1), in biopsies or gonadectomies from male patients with 45,X/46,XY mosaicism. Column 1: expression of INSL3 and CYP11A1 in samples without presence of Leydig cell (LC) hyperplasia. Column 2: expression pattern in areas with LC hyperplasia. Column 3: INSL3 and CYP11A1 expression in areas with severe LC hyperplasia.

In a total of 16 available samples (from 50% of included patients), immunohistochemical staining for INSL3, CYP11A1, and 3β-HSD expression was examined and Leydig cell hyperplasia was quantified (**Table 2**). Overall, there was an overlap in expression between INSL3 and the Leydig cell markers CYP11A1 and 3β-HSD, but with fewer Leydig cells expressing 3β-HSD. Leydig cell areas were quantified based on the CYP11A1-positive cells and was expressed as a percentage of total biopsy areas (TLCA/TSA). The percentage of TLCA/TSA ranged from 0-55% in the included biopsies (**Table 2**). In biopsies with testicular morphology, there was generally a complete overlap between the expression patterns of INSL3 and CYP11A1, except for one prepubertal testis sample in which only CYP11A1 expression was detected in a few cells. Also, in four samples containing areas with severe hyperplasia (all samples with TLCA/TSA≥27%), INSL3-negative/CYP11A1-positive areas were observed (**Figure 3**). Seemingly, there was no link between INSL3 expression and Sertoli-cell only pattern, presence or differentiation of germ cells, or germ cell neoplasia *in situ*.

## Discussion

This pilot study is the first to describe serum concentrations and gonadal expression of INSL3 in male patients with 45,X/46,XY mosaicism and to concomitantly evaluate clinical and histological outcomes. In general, INSL3 concentrations were low in many patients, but elevated in a few. Interestingly, INSL3 expression was lost in a sub-population of Leydig cells in areas with severe hyperplasia. Altogether, there were no observed patterns between serum INSL3 and clinical phenotype nor between INSL3 expression and histological phenotype. However, further prospective studies are needed to ascertain the clinical role of INSL3 in these patients.

Most of the untreated patients included in the study had low-normal serum INSL3 concentrations, although two had elevated concentrations (+2.0 and +2.7 SD scores, respectively). Interestingly, serum testosterone SD scores were either lower, similar to, or higher than the corresponding INSL3 SD scores, but all within the reference range. It has been speculated that INSL3 as a marker of Leydig cell capacity and differentiation is more sensitive than testosterone to pre-, peri- and postnatal testicular development (1). Male patients with 45,X/46,XY mosaicism have been reported to often have fairly normal testicular function (12–16) and, thus, it can be speculated that the relatively normal concentrations of INSL3 reported in the current cohort may reflect comparatively normal testicular development. Moreover, all patients undergoing testosterone treatment had serum INSL3 values below -3 SD scores, which is in line with previous studies of testosterone-treated males with hypogonadism (1, 31–33). Similarly, the prepubertal patient also had undetectable serum INSL3, which was unsurprising given the relatively quiescent state of the Leydig cells at the age of eight years. It can be discussed whether a serum INSL3 concentration from a gonadectomized patient should be included; however, the undetectable concentration verified the performance of the INSL3 method.

The gonadotropins were normal to elevated and LH SD scores were higher than INSL3 and testosterone SD scores in almost half of the untreated patients, which may indicate compensated Leydig cell insufficiency in some patients. The patients with elevated FSH had correspondingly low inhibin B concentrations suggestive of hampered spermatogenesis. Interestingly, AMH concentrations were normal in all untreated patients, which, to our knowledge, is the first report of its kind. It can be speculated that inhibin B is the first of these two Sertoli cell markers to indicate impaired function. Overall, the biochemical findings were supported by the clinical observations: Leydig cell function in terms of external genital phenotype at birth, spontaneous pubertal onset, and the need for testosterone treatment was overall good. Likewise, azoospermia rates were high as reflected in the high FSH/low inhibin B concentrations. This is in line with previous studies on 45,X/46,XY patients, reporting high degrees of external genital androgenization at birth in some patients (13, 16, 34), high rates of spontaneous puberty, some patients not in need of testosterone treatment (12–16), but frequent infertility (12, 17, 23–25). In this study, there was no observed association between serum INSL3 concentrations and external genital androgenization at diagnosis or rates of spontaneous pubertal onset.

Gonadal morphology was overall characterized by varying degrees of testicular dysgenesis in all 16 included samples originating from biopsies and gonadectomies. Leydig cell hyperplasia and focal Sertoli cell-only pattern was present in the majority of samples. In line with these findings, germ cells were only present in approximately half of the samples. These characteristics have also been described in gonadal samples from patients with a 45,X/46,XY karyotype previously (12, 13, 16). However, the focal nature of biopsies was highlighted by the discrepancy observed in the two patients who had spermatozoa in their semen samples, while complete spermatogenesis was not reported in their testicular biopsies. Namely, in these two patients the highest degree of germ cell differentiation was spermatocytes in the evaluated testicular tissue. Complete spermatogenesis in histological evaluations of testicular material from patients with 45,X/46,XY mosaicism has previously been reported (12, 25), but no samples in the current study included spermatozoa. Moreover, in 15% of patients with available information on gonadal histology, germ cell neoplasia *in situ* was found, which is similar to other studies reporting its presence in approximately 10-15% although with some variation (12, 13, 16, 21, 35).

INSL3 expression was present in all examined postpubertal testicular samples and in the majority of Leydig cells positive for the established marker CYP11A1 (9). However, in samples containing areas with severe Leydig cell hyperplasia, a sub-population of the CYP11A1-positive cells did not express INSL3. This pattern has also been reported in testicular samples from patients with human chorionic gonadotropin-producing tumors (9). CYP11A1 is an enzyme that, inter alia, catalyzes the conversion of cholesterol to pregnenolone and thereby contributes to the initiation of steroidogenesis. As such, it could be speculated that as ‘markers’ of the two Leydig cells products (INSL3 and testosterone), the lack of overlap in hyperplastic areas reflects the clinical observation that in some patients INSL3 is relatively lower than testosterone. This is true for the one untreated patient in this cohort without complete immunohistochemical overlap; serum INSL3 was low-normal, while testosterone was high-normal. This possible link between Leydig cell hyperplasia and observed biochemistry may also be true for other hypogonadal patients. For example, in two previous studies that included testicular samples from patients with Klinefelter syndrome, CYP11A1 in one and the steroidogenic acute regulatory protein (StAR, i.e. the initiator of steroidogenesis catalyzed by CYP11A1) in the other, were expressed in all Leydig cells, while INSL3 was not (9, 11). The clinical discrepancy between low serum INSL3 and relatively higher testosterone has also been observed by our group in untreated patients with Klinefelter syndrome (36).

With the inclusion of both serum INSL3, gonadal expression of INSL3, and clinical and histological parameters, an aim of this study was to describe INSL3 *in toto*. However, there were no observed links between serum INSL3 and the clinical outcomes nor between INSL3 expression and histological outcomes. Furthermore, the very high serum INSL3 values in two of the patients were perplexing, but could theoretically reflect a compensatory rescue mechanism due to the risk of germ cell apoptosis. Studies in rats have indicated that INSL3 may anti-apoptotic qualities, for example *via* the presence of the INSL3 receptor on the seminiferous tubules and on selected stages of germ cells (4) and *via* treatment of rats with e.g. INSL3 and gonadotropin-releasing hormone, and/or an INSL3 antagonist (4, 5). While high serum INSL3 in hypogonadal patients may indicate germ cell sparring mechanisms, low serum INSL3 may indicate germ cell loss. However, we observed no link between serum INSL3 and the presence of germ cells or spermatogenesis/spermatozoa in semen samples in this study. It is important to note that the serum sampling was not performed at the time of the biopsies/gonadectomies.

The strengths of this study included: 1) eighteen patients with a 45,X/46,XY karyotype with detailed clinical, biochemical, and histological descriptions; 2) all samples were measured with the same highly sensitive INSL3 analysis based on LC-MS/MS methodology; and 3) all hormone values were expressed as SD scores allowing for comparisons across all ages. However, this study also included limitations: 1) the study group was small and existing associations may be overlooked; 2) the retrospective design caused missing clinical and histological data for some patients and two without serum INSL3 measurements; and 3) the time lapse between serum sampling and clinical and paraclinical (semen and histological samples) may influence possible conclusions on the potential use of serum INSL3 as a gonadal marker in 45,X/46,XY mosaicism.

In conclusion, this pilot study was the first to evaluate serum concentrations and gonadal expression of INSL3 in males with a 45,X/46,XY karyotype. Serum INSL3 concentrations spanned from below to above the reference range and seemed independent of other reproductive hormones and clinical features. Immunohistochemical staining of INSL3 and CYP11A1 were discordant in areas with severe hyperplasia and may explain why some hypogonadal patients have low INSL3 concentrations despite normal testosterone concentrations. However, the clinical role of INSL3 remains unclear and further, larger studies are warranted.

## Data Availability Statement

The original contributions presented in the study are included in the article/supplementary material. Further inquiries can be directed to the corresponding author.

## Ethics Statement

The studies involving human participants were reviewed and approved by The Regional Ethics Committee, Capital Region of Denmark (H-1-2012-007). Written informed consent from the participants’ legal guardian/next of kin was not required to participate in this study in accordance with the national legislation and the institutional requirements. All included serum and histological samples were retrieved as part of clinical follow-up. Extraction and management of patient data were approved by the Danish Patient Safety Authority (no. 3-3013-1376/1/) and the Danish Data Protection Agency (no. 2015-235, I-Suite no. 04204). Immunohistochemical staining of gonadal tissue was approved by the local ethics committee (H-1-2012-007). INSL3 measurements were approved as part of a quality assurance project at Rigshospitalet, University Hospital of Copenhagen (no. 20012618).

## Author Contributions

MLL, AJø, and THJ took part in the study design, execution, analysis, manuscript drafting and critical discussion. LA, JN, and JA took part in the execution, manuscript drafting, and critical discussion. AJu took part in the analysis, manuscript drafting, and critical discussion. All authors contributed to the article and approved the submitted version.

## Funding

This work was supported by The Absalon Foundation (MLL), The Innovation Fund Denmark (14-2013-4) (AJu, JA), The ReproUnion Collaboration (AJu, JA), and The European Union, Interreg V ÖKS (NYPS-ID 20200407) (AJu, JA).

## Conflict of Interest

The authors declare that the research was conducted in the absence of any commercial or financial relationships that could be construed as a potential conflict of interest.

## Publisher’s Note

All claims expressed in this article are solely those of the authors and do not necessarily represent those of their affiliated organizations, or those of the publisher, the editors and the reviewers. Any product that may be evaluated in this article, or claim that may be made by its manufacturer, is not guaranteed or endorsed by the publisher.
